# Mapping main, epistatic and sex-specific QTL for body composition in a chicken population divergently selected for low or high growth rate

**DOI:** 10.1186/1471-2164-11-107

**Published:** 2010-02-11

**Authors:** Georgina A Ankra-Badu, Daniel Shriner, Elisabeth Le Bihan-Duval, Sandrine Mignon-Grasteau, Frédérique Pitel, Catherine Beaumont, Michel J Duclos, Jean Simon, Tom E Porter, Alain Vignal, Larry A Cogburn, David B Allison, Nengjun Yi, Samuel E Aggrey

**Affiliations:** 1 Department of Biostatistics, Section on Statistical Genetics, University of Alabama at Birmingham, Birmingham, AL 35294, USA; 2 National Institutes of Health/The National Human Genome Research Institute, Bethesda, MD 20892, USA; 3 Institut Nationale de la Recherche Agronomique, UR83 Recherche Avicoles, F-37380 Nouzilly, France; 4 INRA, Laboratoire de Genetique Cellulaire, 31326 Castanet-Tolosan, France; 5 Department of Animal and Avian Sciences, University of Maryland, College Park, MD 20742, USA; 6 Department of Animal and Food Sciences, University of Delaware, Newark, DE 19717, USA; 7 Clinical Nutrition Research Center, University of Alabama, Birmingham, AL 35294, USA; 8 Department of Poultry Science/Institute of Bioinformatics, University of Georgia, Athens, GA 30602, USA

## Abstract

**Background:**

Delineating the genetic basis of body composition is important to agriculture and medicine. In addition, the incorporation of gene-gene interactions in the statistical model provides further insight into the genetic factors that underlie body composition traits. We used Bayesian model selection to comprehensively map main, epistatic and sex-specific QTL in an F_2 _reciprocal intercross between two chicken lines divergently selected for high or low growth rate.

**Results:**

We identified 17 QTL with main effects across 13 chromosomes and several sex-specific and sex-antagonistic QTL for breast meat yield, thigh + drumstick yield and abdominal fatness. Different sets of QTL were found for both breast muscles [*Pectoralis (P) major *and *P. minor*], which suggests that they could be controlled by different regulatory mechanisms. Significant interactions of QTL by sex allowed detection of sex-specific and sex-antagonistic QTL for body composition and abdominal fat. We found several female-specific *P. major *QTL and sex-antagonistic *P. minor *and abdominal fatness QTL. Also, several QTL on different chromosomes interact with each other to affect body composition and abdominal fatness.

**Conclusions:**

The detection of main effects, epistasis and sex-dimorphic QTL suggest complex genetic regulation of somatic growth. An understanding of such regulatory mechanisms is key to mapping specific genes that underlie QTL controlling somatic growth in an avian model.

## Background

A clear understanding of the genetic architecture of body composition is important in chicken breeding. Genetic selection over the past 50 years has produced commercial meat-type (broiler) chickens with a higher yield of breast meat, which is also accompanied by increased body fatness [1]. Breast muscle yield is the most important carcass component in meat-type chickens because of the high premium paid by consumers. However, excess accumulation of body fat is undesirable because it reduces the efficiency of feed utilization and it adds the additional expense of trimming unwanted fat during processing [2]. Higher consumption of excess dietary saturated fat contributes to artherosclerosis in humans. Therefore delineating major genes that underlie carcass traits has important implications for both agriculture and human health.

Crosses from extreme strains and/or breeds of chickens have been used to map quantitative trait loci (QTL) for body composition traits including fatness [3-9]. Despite the importance of dissecting the genetic basis of body composition in the chicken, body composition QTL mapping studies to date have been restricted to delineating the main (additive and dominance) genetic effects. Incorporation of epistasis and sex-dimorphism into QTL analyses has the potential to identify novel epistasis QTL, and sex-specific and sex-antagonistic QTL [10,11]. Physiological differences between sexes can influence gene expression [12]. Therefore, sex-specific QTL are to be expected for carcass traits, especially abdominal fatness. Abasht et al. [7] have mapped an abdominal fatness QTL on chicken chromosome 5 (GGA5) that exhibits sexual dimorphism. Furthermore, several sex-specific QTL for body composition have been reported in humans and in rodent models [13,14].

Empirical evidence suggests that fat and body composition traits are influenced by epistasis [11,15]. Earlier studies by Carlborg et al. [16] and Yi et al. [11,17,18] have demonstrated that joint assessment of regions on the genome, on either the same chromosome or different chromosomes have significant effects on traits. Therefore, inclusion of gene-gene interactions in the statistical model is essential in providing comprehensive mapping of the genetic factors that underlie body composition traits. Modeling of these gene interactions has been challenging because of the large number of variables [19] and the decreased power of the statistical analysis [20]. These drawbacks can be ameliorated by utilizing a Bayesian model selection method which models main, epistatic and gene-environmental effects simultaneously [19]. The Bayesian approach has been used successfully to identify several epistatic QTL associated with growth and body composition in mice [11,18].

Herein, we used Yi et al.'s [21] Bayesian model selection method to comprehensively investigate main, sex-specific and gene-gene interaction effects of body composition traits in a chicken population divergently selected for high or low growth rate.

## Results

### Main effect QTL

**Figure 1:**
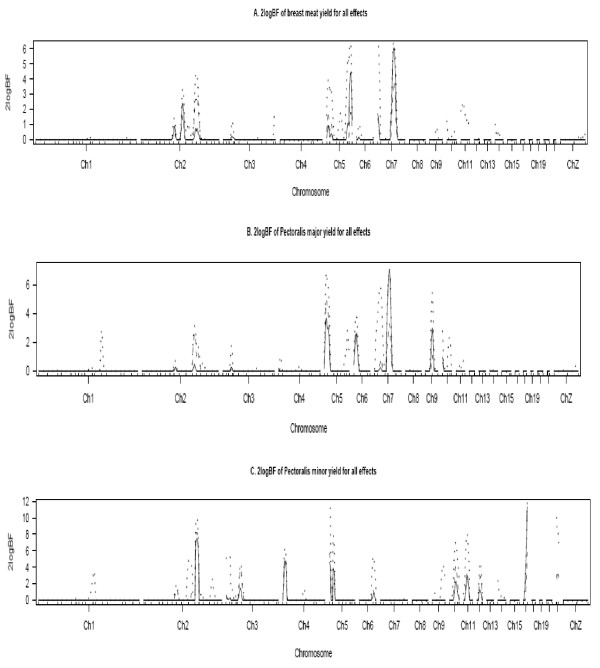
**One-dimensional profiles of Bayes factors rescaled as 2log_e _BF for main (solid lines), epistatic effects (dotted lines) and sex-specific effects (dashed lines)**. **A**: Breast meat yield **B**: *Pectoralis *(*P*) *major ***C**. *P. minor *yield. The horizontal lines represent the significance threshold of 2log_e_BF = 2.1.

The trait means and standard deviations for the F_2 _resource population are presented in Table 1. The main-effect QTL affecting carcass traits are summarized in Table 2. Seventeen QTL with significant linkages were observed on 13 chromosomes (Figure 1). A QTL for the *Pectoralis (P) major *weight was found on GGA7 at 87 cM and for *Pectoralis minor *weight on GGA3, 4 and 17. Nevertheless, adjusting *P. minor *for BW at 9 wk only confirmed the QTL on GGA4 and 17. A thigh + drumstick QTL was located on GGA27 at 0 cM. Seven QTL for ABFW were identified on GGA1, 2, 5, 7, 14, 15 and 18. Adjusting ABFW with BW at 9 wk revealed additional QTL for ABFY on GGA1, 3, 5, 9, 12, and 27. The TDW and TDY QTL co-localized with an ABFY QTL on GGA27. The QTL effect in terms of genotypic mean placements and the proportion of the phenotypic variance explained are also shown in Table 2. The main effect QTL explained from 1 to 14% of the phenotypic variance. The *P. major *and *P. minor *yields QTL on GGA7 and GGA17, each explained about 6% of the phenotypic variance. The ABFW QTL on GGA5 and GGA7 contributes ~27% of the phenotypic variance.

**Table 1:** Body composition traits of F_2 _individuals from F_1 _crosses of divergent chicken lines selected for high or low growth (mean ± standard deviation)

Trait	Male (N = 371)	Female (N = 324)
Breast meat weight, g	69.22 ± 9.54	58.16 ± 8.96
Breast meat yield, %	5.57 ± 0.41	5.82 ± 0.48
Abdominal fat weight, g	18.86 ± 11.29	18.52 ± 11.24
Abdominal fat yield, %	1.48 ± 0.80	1.80 ± 0.98
*Pectoralis major *weight, g	51.24 ± 7.16	42.86 ± 6.77
*Pectoralis major *yield, %	4.12 ± 0.32	4.29 ± 0.37
*Pectoralis minor *weight, g	17.94 ± 2.73	15.30 ± 2.45
*Pectoralis minor *yield, %	1.44 ± 0.14	1.53 ± 0.15
Thigh + drumstick weight, g	142.16 ± 18.10	109.48 ± 16.05
Thigh + drumstick yield, %	11.43 ± 0.39	10.97 ± 0.46

**Table 2:** Main QTL effects, location and phenotypic variance explained by body composition traits in chicken lines divergently selected for low or high growth for combined sex.

Chromosome	QTL Position (cM)	2Log BF^1^	Effect^2^	Variance explained by QTL effect
Breast meat yield				
7	87.0	6.77	49.17	3.01
*Pectoralis major *weight				
7	87.0	6.48	46.34	3.02
*Pectoralis major *yield				
7	83.0	8.07	80.13	7.00
*Pectoralis minor *weight				
3	78.7	2.21	-23.55	1.09
4	14.0	4.02	28.66	1.08
17	21.0	11.08	39.59	6.63
*Pectoralis minor *yield				
4	12.0	4.69	30.62	1.64
17	21.0	11.30	38.11	6.17
Thigh + drumstick weight				
27	0.0	10.17	32.19	1.16
Thigh + drumstick yield				
27	0.0	10.73	24.55	1.34
Abdominal fat weight				
1	202.0	7.25	154.38	6.62
2	276.0	6.66	-217.64	4.03
5	101.4	8.63	-28.23	13.26
7	2.1	11.13	128.13	13.74
14	16.4	6.10	69.83	7.08
15	30.8	5.39	-29.93	5.09
18	14.9	9.34	-42.46	3.80
Abdominal fat yield				
1	424.5	4.04	47.29	2.26
3	76.6	4.56	-21.28	1.10
5	10.0	6.69	0.66	4.28
9	12.0	3.95	27.06	1.71
12	18.7	3.45	4.33	1.05
27	0.0	8.26	14.04	1.97

^1 ^Twice the log of the Bayes Factor ^2^Main effect of the QTL

### QTL × Sex interactions

The presence of significant QTL by sex interactions in the model for all parameters presently considered suggested either sex specificity or sex-influenced QTL. We identified several sex-specific and sex-antagonistic QTL which are listed in Supplementary 1. Sexual dimorphism was observed for both *P. major *and *P. minor *yield on GGA2 and 5. Whereas, all QTL detected for *P. major *were female-specific, both male-and female-specific QTL were identified for *P. minor *QTL. Multiple QTL were detected on the same chromosome in some cases, but for different sexes. For example, a male-specific *P. minor *yield QTL was detected on GGA5 at 0 cM, whereas, the female- specific QTL was detected at 12 cM. The phenotypic variances explained by these sex-specific QTL ranged from 1 to 27%.

### Epistatic effects

**Figure 2:**
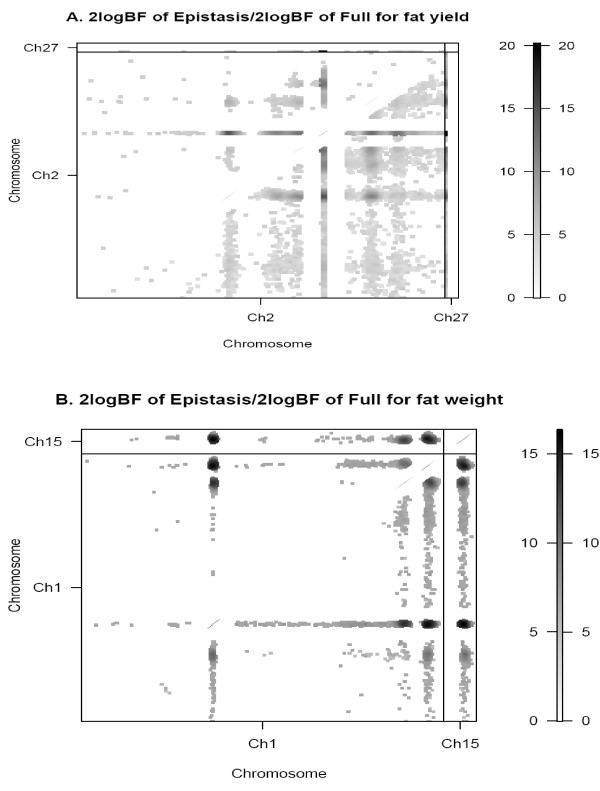
**Two-dimensional profiles of Bayes factors (rescaled as 2logeBF) for fat yield (Figure 2A) and fat weight (Figure 2B) for selected chromosomes**. The upper diagonal shows the Bayes factor for the epistatic model, the lower diagonal shows the Bayes factor for the full model with epistasis compared with no QTL.

Significant QTL by QTL interactions were found for both muscle yield and abdominal fatness traits. Each epistatic QTL explained ~3 to 25% (Table 3) of the phenotypic variance. A highly significant epistatic QTL (2logBF ~17.49; GGA2/6) for ABFY explained approximately 25% of the phenotypic variance. A region on GGA2 (284-293 cM) interacts with GGA1, 6 and 27 and explains a large (~12 to 25%) proportion of the phenotypic variance for ABFY. The same position on GGA2 interacts with other regions of the genome to influence *P. major *weight and yield. In addition to inter-chromosomal interactions, an intra-chromosomal interaction affecting *P. major *yield was found between positions 33.4 and 83.0 cM on GGA7. Significant interactions were found between QTL for ABFY on GGA1 and 2 (Figure 2A). Fat weight on the other hand was strongly influenced by interactions between GGA1 and 1, GGA1 and18, GGA1 and 15, GGA1 and 18, and GGA2 and 18 (Figure 2B).

**Table 3:** Epistatic QTL effects, locations and phenotypic variance explained for body composition traits in a chicken line divergently selected for low or high growth

Chromosome	Interacting QTL positions	2LogBF	Epistatic Effect	Variance explained by Epistatic QTL
Breast meat yield				
2/5	331.6/86.8	9.49	165.00	3.92
2/7	256.0/101.0	10.54	251.00	3.34
5/7	132.5/89.0	13.36	148.00	5.57
7/7	62.3/91.0	14.12	113.10	10.01
11/11	6.2/16.5	9.31	129.50	4.54
*Pectoralis major *weight				
2/3	315.0/70.4	8.34	209.70	3.39
2/5	329.0/82.6	9.30	157.20	4.70
2/7	307.0/133.0	9.95	276.20	3.98
5/7	107.7/72.9	12.78	157.00	6.58
7/7	39.6/77.0	13.95	100.30	7.28
*Pectoralis major *yield				
2/2	284.0/313.0	6.80	205.00	3.17
2/5	292.4/63.6	8.63	143.00	3.32
5/7	20.1/68.6	13.92	81.00	8.29
5/9	30.4/40.1	12.25	36.00	2.81
7/7	39.6/68.6	12.93	103.00	8.16
*Pectoralis minor *weight				
2/5	290.3/14.0	20.16	109.20	7.63
2/11	290.3/41.0	16.88	60.26	8.52
5/11	69.9/14.4	17.49	35.80	4.09
10/17	42.0/6.3	15.84	28.40	3.20
11/17	26.8/21.0	17.75	21.05	4.98
*Pectoralis minor *yield				
2/4	294.5/14.0	15.47	119.59	7.60
2/5	288.2/2.0	20.32	94.30	7.70
2/17	294.5/21.0	19.82	123.23	6.69
5/11	10.0/18.5	17.88	26.77	2.85
11/17	22.6/21.0	18.06	16.96	5.69
Thigh + drumstick weight				
1/1	40.9/436.7	13.12	264.50	6.80
1/5	42.9/65.7	12.70	135.00	6.69
1/27	106.0/0.0	17.10	161.60	8.11
3/5	266.7/48.9	12.89	144.10	3.55
7/27	151.0/0.0	19.98	68.10	3.94
Thigh + drumstick yield				
1/1	47.0/416.4	13.57	264.21	6.73
1/7	128.0/6.4	15.67	127.74	5.64
1/27	104.0/0.0	17.52	101.12	8.09
2/7	91.0/6.4	10.33	127.74	4.68
10/27	53.0/0.0	16.74	9.52	4.23
Fat weight				
1/1	196.0/529.2	14.16	522.00	10.62
1/11	210.2/39.0	9.48	469.00	7.80
1/15	206.0/20.5	14.1	561.50	12.26
1/18	194.0/10.6	6.18	511.40	16.60
2/18	286.1/14.9	6.84	492.30	10.81
Fat yield				
1/2	229.0/284.0	16.37	98.97	17.11
2/2	192.0/288.0	14.87	89.41	17.43
2/6	292.4/73.0	17.49	82.27	24.73
2/27	292.4/0.0	20.15	1.68	12.06
5/6	20.1/75.0	16.17	49.50	11.01

## Discussion

Most traits of economic and biomedical importance are influenced by multiple genetic and environmental factors. Using techniques that allow for inclusion of epistasis and sex-specificity in a QTL model enables a better understanding of the genetic regulatory mechanisms that underlie body composition. Chicken breast muscle is comprised of two distinct muscle groups: the *P. major *and the *P. minor*. A significant main- effect QTL for BMY was detected on GGA7; yet analysis of each breast muscle indicates that *P. major *was the only trait contributing to BMY on GGA7. The location of the *P. major *QTL is similar to that reported for BMY [9,22]. The QTL region for *P. major *yield contains several genes [the interferon induced with helicase C domain 1 (*IFIH1*), glucagon (*GCG*), ring finger protein 25 (*RNF25*) and BAX inhibitor motif containing 1 (*TMBIM1*)]. We identified three novel QTL for *P. minor *weight on GGA3, 4 and 17; however after adjustment with BW at 9 wk, the QTL on GGA4 and 17 met the level of significance. The *P. minor *yield QTL on GGA17 explained approximately 6% of the phenotypic variation. The pre-B-cell leukemia transcription factor 3 (*PBX3*), a homeobox gene is located within this QTL region. Most studies evaluate breast meat as a single trait [8,23,24]. The current study suggests that these traits should be treated independently since they are influenced by different QTL. We also identified a QTL for TDW and TDY at the same location (0 cM) on GGA27. The 0 cM region of GGA27 harbors the mitogenic activated protein kinase kinase 14 (*MAP3K14*), defender against cell death 1 (*DAD1*) and MYST histone acetyltransferase 2 (*MYST2*) genes.

Intensive genetic selection of meat-type chicken during the the last 50 years has led to rapid somatic (muscle) growth and a concomitant increase in ABFY [25]. Abdominal fatness is a complex trait affected both by genes, environmental factors (nutrition, appetite, behavior, etc), and their interactions. In the present study, we found QTL for ABFW on GGA1, 2, 5, 7, 14, 15 and 18. The QTL for ABFW on GGA1, 5, 15 and 18 were similar to the location reported for these traits in other chicken populations [4,6,22]. When ABFW was corrected for BW at 9 wk, ABFY QTL were confirmed on GGA1 and GGA5, and novel ones were identified on GGA3, 9, 12 and 27. The ABFY QTL on GGA27 co-localized with the TDY QTL, while the position of the ABFY QTL on GGA3 is similar to a suggested fatness QTL (10% chromosome-wide significance) by Lagarrigue et al. [18]. The ABFY QTL on GGA1 harbors thyroid hormone responsive protein (*THRSP*) which is a nuclear protein expressed in lipogenic tissues (liver, fat and lactating mammary glands), and is involved in the transduction of hormonal and dietary signals for increased lipid metabolism [26]. The *THRSP *gene is differentially expressed in the high and low lines; and mutants of THRSPα are associated with ABF in chickens [27]. The THRSP gene also modulates tumorigenesis in human breast cancer [28]. Positional candidate genes that underlie the GGA3 ABFY QTL include inhibitor of growth, family member 1 (*ING1*), Rho guanine nucleotide exchange factor 7 (*ARHGEF7*) and ankyrin repeat domain 10 (*ANKRD10*). The ABFY QTL on GGA5 harbors the insulin gene and insulin-like growth factor 2 (*IGF2*) gene. A biallelic marker in the chicken *IGF2 *gene appears to be associated with growth and carcass traits [29].

### Sex-specific QTL

Several studies in other species point to sex-bias, sex specificity or sex antagonism in QTL analysis [30-32]. The approach allows us to test for interactions between QTL and sex. A QTL by sex interaction with a Bayes Factor (2LogBF) ≥ 2.1 was considered as sex specific (QTL influencing a trait in only one sex) or sex antagonistic (QTL with allelic effects going in opposite directions between the sexes). A sex antagonistic fatness QTL has been reported in chickens divergently selected for abdominal fatness [7]. Sex-antagonistic QTL for ABFY were found on GGA2, 4, 6, 12, 14 and 19. Male-specific QTL for ABFY on GGA2 and GGA4 were similar to those reported by Jennen et al. [4] and McElroy et al. [6], respectively. However, the ABFY QTL on GGA6, 12, 14 and 19 (Additional file 1) are unique to the HG × LG cross. There were several female-specific QTL affecting *P. major *yield, and contrarily several sex-antagonistic QTL affecting *P. minor *yield. Some fatness QTL were also found to be sex-antagonistic in the current study. The male-specific QTL for ABFW was within the confidence interval of the sex-antagonistic QTL for abdominal fatness reported by Abasht et al. [7]. Sex-specific QTL and their genetic inter-relationships have been reported for human obesity and lipid levels [14]. The mechanisms underlying sex-specific, sex influenced or sex-antagonistic effects are unknown although the influence of sex hormones on the regulation of the genes that underlie these QTL is the first evident hypothesis. Other parameters showing sex-dimorphism (such as food intake, plasma nutrient levels etc.) may exert further additional controls on their own. The fine mapping strategies utilized to identify major genes that underlie QTL would depend on whether QTL effect is additive, epistatic, sex-specific or sex-antagonistic.

### Epistatic QTL effects

By definition, a complex trait is affected by many genes, each with a small effect, the environment and gene by environment interactions. However, in most instances the summation of the additive effects of each single-locus cannot explain all the phenotypic variation of a particular trait. The dependency of one locus upon another, referred to as epistasis, also contributes towards the phenotypic variation. The inclusion of epistatic effects through interactions of different QTL regions (same or different chromosomes) in QTL mapping allows for the detection of novel loci. Epistatic QTL explained between 3 to 25% of the phenotypic variation. Epistasis QTL involving positions on GGA 1, 2, 3, 4, 5, 6, 7, 9, 10, 11, 17 and 27 were associated with body composition traits in the current study. An earlier study utilizing a White Leghorn × Red Jungle fowl cross identified many epistatic pairs that affected both early and late growth [16]. They argued that, the degree of divergence between their populations could be the reason for the measured epistasis. Gene interactions may be the norm rather than the exception. Limited studies on epistasis QTL are due principally to the lack of statistical methods with sufficient power to detect them, rather than their lack of existence. Other studies have described the effect of epistasis on fatness in mice [11,17,18,33,34]. Genes that underlie interacting QTL may interact biologically or may code for enzymes involved in common pathways [35]. Several positional candidate genes at the GGA2 284-286.1 cM region [Yamaguchi sarcoma viral oncogene homolog 1 (*YES1*), GATA-6-transcription factor (*GATA-6*), retinoblastoma binding protein 8 (*RBBP8*), Rho-associated, coiled-coil containing protein kinase 1 (*ROCK1*)] could be interacting with other genes on GGA6 and 27 to affect abdominal fatness in meat-type chickens. It appears that some of the candidate genes that underlie QTL for ABFY are also associated with breast cancer in humans [28]. Therefore candidate genes within the QTL regions identified in this study should be investigated for their biologically significance to body composition in chickens and to obesity and cancers in humans.

## Conclusions

We have studied the main genetic and interactive effects of trait loci that affect body composition in chickens. Our studies have confirmed some known QTL http://www.animalgenome.org/QTLdb/chicken.html and identified some novel QTL in the high and low growth line intercross. The Bayesian statistical strategy has allowed us to concurrently explore epistatic, sex-specific and sex-antagonistic QTL. Identification of genes that underlie QTL regions and their interactions as demonstrated by significant QTL-QTL interactions should provide insight into an elaborate network of genes and will help to elucidate their role in body composition and fatness in chickens and possibly breast cancer in humans.

## Methods

### Experimental population

An F_2 _population was generated by inter-mating two experimental boiler lines that had been divergently selected for high (HG) or low growth (LG) rate [36]. In the F0 generation, five HG males were mated to 16 LG females (HL) and 5 LG males to 9 HG females (LH). From the F_1 _generation, 3 HL males were mated with 30 HL females and 2 LH males were intercrossed with 20 LH females to generate an F_2 _resource population of 695 (371 males and 324 females) F_2 _individuals. The F_2 _population was produced in four hatches, fed a standard broiler diets *ad libitum *(3050 kcal ME (Metabolizable energy) from 0-3 wks, 3100 kcal ME from 4-9 wks), and raised under standard management practices for nine weeks. Blood was taken from all birds for genomic DNA extraction. At 9-wk, birds were weighed after an overnight fast and slaughtered. After evisceration, carcasses were stored overnight at 4°C before dissection. The carcass traits measured are breast meat weight (BMW) and yield (BMY), and its two components: *P major *and *P. minor *weights, abdominal fat weight (ABFW) and thigh + drumstick weight (TDW). Trait weights were corrected for week 9 body weight to generate *P. major *yield, *P. major *yield, ABF yield (ABFY) and thigh + drumstick yield (TDY).

### Genotyping

DNA was extracted from whole blood by a quick preparation method [8]. Microsatellite markers were selected from the poultry genetic consensus map [37] based on chromosomal locations and informativeness in each F_0 _sire family. The platform used for genotyping was developed at the Centre de Resources, Génotypage, Séquençage (CRGS) of Génopole Toulouse Midi-Pyrenées (INRA, Toulouse, France). Genotyping of the DNA samples employed 109 informative markers representing 20 autosomal linkage groups and was performed at Labogena (INRA Jouy-en-Josas, France). Fluorescent microsatellite analysis was performed on ABI 3700 DNA sequencers (Applied Biosystems, Foster City, CA). Each genotype was interpreted using both the GeneScan Analysis 3.7 and Genotyper Analysis 3.7 software (Applied Biosystems, Foster City, CA). The GEMMA database was used to manage the informativeness of the genotyping assays [38].

### Data reformatting

The multi-allelic nature of microsatellite markers required formatting to determine the F_0 _line of origin of each marker. Using the F_2 _coding format from the R/qtl software [39], the genotype of each individual at each marker was coded as follows: AA (if the both alleles were inherited from HG grandparents), AB (if the alleles were derived from one HG and one LG grandparent), BB (if both alleles were inherited from two LG grandparents), not BB (if one allele was inherited from an HG parent and the origin of the other allele was indeterminate), not AA (if one allele was inherited from an LG parent and the origin of the other allele was indeterminate) and NA (if the line of origin could not be determined for either allele).

### Statistical analysis

Yi et al.'s [18,21] Bayesian model selection method was used to simultaneously detect main effects and epistatic and gene-sex interactions using the R/qtlbim software [40]. Each chromosome was divided into one cM grids, resulting in 2410 possible loci across the chicken genome. These preset loci were considered as possible QTL positions. We placed an upper bound on the number of QTL included in the model for each trait. The upper bound was chosen based on the number of significant QTL detected in the traditional interval mapping [21]. Based on this, the prior number of main-effect QTL was set at *l*_m _and the prior for the expected number of all QTL was *l*_m _+ 3. We simultaneously modelled main effects, QTL-QTL interactions and QTL-sex interactions. We fitted the models using R/qtlbim [40], which implements a Markov chain Monte Carlo (MCMC) algorithm [18,21]. The MCMC algorithm generates posterior samples from the joint posterior distribution of all parameters in the model, proceeding to draw each parameter from its conditional posterior distribution using the latest values of all other unknowns and the observed data. Each iteration of the MCMC algorithm cycles through all elements of the unknowns. This process was continued for many iterations in order to obtain random samples from the joint posterior distribution. For each analysis, the MCMC sampler was run for 1.2 × 10^5 ^iterations after the first 1000 iterations were discarded as burn-in. To reduce serial correlation in the stored samples, the chain was thinned by one in *k *= 40, yielding 3 × 10^3 ^samples for posterior analysis. Convergence diagnostics and mixing behavior assessed using graphical and numerical methods provided by R/qtlbim showed that the simulation chains converged and mixed well.

The posterior inclusion probability for each locus was estimated as its frequency in the posterior samples. Each locus may be included in the model through its main effects and/or interactions with other loci (epistasis). The larger the effect size for a locus, the more frequently the locus was sampled. Taking the prior probability into consideration, we used the Bayes factor (BF) to show evidence for inclusion versus exclusion of a locus. The BF for a locus is defined as the ratio of the posterior odds to the prior odds for inclusion versus exclusion of the locus. A BF threshold of 3, or 2log_e _(BF) = 2.1, is taken as supporting a claim of significance [41]. The posterior inclusion probability and corresponding BFs of main effects, epistatic interactions and QTL-sex interactions were estimated separately. The proportions of the phenotypic variance explained by the genetic effect were estimated by its heritability.

## Authors' contributions

GB carried out the QTL mapping analyses and drafted the manuscript. DS, NY helped in the programming and interpretation of the data. FP and AV supervised the genotyping of the study. SG, CB, MD, JS, TP, ED, TP, LC participated in the design of the study and data collection and helped to revise the manuscript. DA helped with the revision of the manuscript. SA supervised the study, participated in the design and final editing. All authors read and approved the final manuscript

## Supplementary Material

Additional file 1 Sex-specific QTL effect, location and phenotypic variance explained by the QTL for body composition traits in a chicken line divergently selected for low or high growth. Click here for file
