# Supplementary material for: Mapping main, epistatic and sex-specific QTL for body composition in a chicken population divergently selected for low or high growth rate
Source: BMC Genomics. 2010 Feb 11;11:107. doi: 10.1186/1471-2164-11-107 (PMC2830984; doi:10.1186/1471-2164-11-107)
Supplement: Additional file 1 — Sex-specific QTL effect, location and phenotypic variance explained by the QTL for body composition traits in a chicken line divergently selected for low or high growth. [file 1471-2164-11-107-S1.DOC]

| Chromosome | QTL location  (cM) | Sex specificity | 2LogBF | Effect | Variance explained by QTL effect |
| --- | --- | --- | --- | --- | --- |
| Breast meat yield  3  6  7  9  *Pectoralis major* weight  1  2  3  5  6  7  9  *Pectoralis major* yield  2  7  9  12  27  *Pectoralis minor* weight  2  5  5  7  9  10  10  11  14  17  *Pectoralis minor* yield  1  2  2  3  5  5  6  7  10  10  11  12  14  17  Thigh + drumstick weight  1  1  3  4  5  7  10  11  Thigh + drumstick yield  1  1  3  3  4  5  7  10  Fat weight  5  7  7  9  15  18  Fat yield  2  2  4  4  6  12  14  19 | 289.0  18.5  107.0  8.0  106.0  290.3  57.5  14.0  18.5  95.0  14.0  298.6  95.0  0.0  31.0  0.0  302.8  0.0  12.0  133.0  14.0  42.0  53.0  26.8  24.6  21.0  307.0  286.0  294.5  29.8  0.0  14.0  83.0  129.0  44.2  53.0  24.7  31.0  0.0  21.0  150.0  196.0  42.5  119.0  10.0  151.0  0.0  49.0  150.0  198.0  42.5  105.0  119.0  10.0  151.0  0.0  99.3  97.0  103.0  10.0  34.9  12.8  292.4  382.7  121.0  233.0  79.0  31.0  0.0  0.0 | Female  Female  Female  Female  Female  Female  Female  Female  Female  Female  Female  Female  Female  Female  Female  Female  Male  Male  Female  Male  Female  Male  Female  Male  Female  Male  Male  Female  Male  Male  Male  Female  Female  Male  Male  Female  Male  Female  Female  Male  Male  Female  Female  Female  Female  Male  Male  Male  Male  Female  Female  Male  Female  Female  Male  Male  Male  Male  Female  Female  Male  Male  Female  Male  Female  Male  Female  Female  Female  Male | 9.98  7.20  5.81  7.48  5.93  7.60  10.46  10.42  7.04  5.35  8.59  8.10  7.58  8.10  8.12  6.27  5.91  12.17  11.73  7.49  9.15  10.36  22.70  7.36  7.00  18.04  4.03  8.13  8.57  16.55  10.12  7.65  10.97  6.86  9.63  20.18  7.55  16.55  10.59  21.0  7.61  4.43  7.40  5.92  7.80  11.82  12.89  3.89  8.13  4.61  7.45  2.65  5.08  7.64  13.37  14.66  11.77  4.76  3.90  7.41  4.54  10.36  7.02  5.37  3.80  5.79  10.43  11.37  6.91  3.93 | 9.83  -19.58  -16.06  42.09  -5.09  2.52  -37.83  54.18  -26.26  -11.89  48.75  -40.65  -73.58  50.50  -5.07  4.33  6.20  10.71  -25.93  -0.55  28.63  -19.36  41.30  -11.52  -19.38  33.13  13.23  -12.39  16.45  -7.46  7.24  16.39  60.14  -7.06  -13.64  54.29  -14.49  21.85  -25.54  48.35  85.33  -92.76  28.30  7.46  26.27  -38.26  -59.53  -19.91  85.42  -90.49  22.15  -71.06  4.06  18.15  -34.71  -54.28  207.85  -30.14  -32.26  29.65  -48.23  208.97  -9.84  0.49  -3.04  0.26  59.76  14.13  -23.29  0.60 | 1.60  1.24  4.52  10.94  5.93  7.60  8.37  17.50  2.09  3.83  8.99  10.11  26.74  15.70  1.00  1.48  2.42  6.32  12.59  1.99  16.78  18.14  23.56  4.11  10.68  5.25  8.73  3.13  6.64  14.24  4.46  5.92  10.81  3.75  12.09  14.03  6.06  6.70  12.23  16.30  8.96  8.37  3.01  2.23  1.73  8.06  6.51  1.52  12.08  8.38  2.79  5.39  1.54  1.26  8.90  6.48  8.66  2.96  9.78  4.68  2.38  4.38  3.33  3.88  1.47  3.10  11.95  5.93  11.29  2.02 |
